# Supplementary material for: Proteostasis collapse, a hallmark of aging, hinders the chaperone-Start network and arrests cells in G1
Source: eLife. 2019 Sep 13;8:e48240. doi: 10.7554/eLife.48240 (PMC6744273; doi:10.7554/eLife.48240)
Supplement: Supplementary file 1. [file elife-48240-supp1.docx]

**Supplementary file 1.** Parameter modifications to simulate different genotypes or relevant physiological conditions.

| **Reaction name** | **Chemical equation^1^** |
| --- | --- |
| Unfolded protein-chaperone binding | Chap + Prot -> ChapProt |
| Protein folding | ChapProt -> Chap + ProtF |
| Protein misfolding | Prot -> ProtM |
| Misfolded protein refolding | ChapM -> Chap + ProtF |
| Chaperone-misfolded protein binding | ProtM + Chap -> ChapM |
| Dimerization (fast) | 2*Prot +Dimer -> 2*Dimer |
| Dimerization (slow) | 2*Prot -> Dimer |
| Dimerization with hexamer | 2*ProtM + Hexamer -> Dimer + Hexamer |
| Dimerization with chaperone and hexamer | 2*ProtM + ChapHexamer -> Dimer + ChapHexamer |
| Chaperone-dimer binding | 2*Chap + Dimer -> ChapDimer |
| Dimer refolding | ChapDimer -> 2*Chap + 2*ProtF |
| Nucleation | 3*Dimer -> Hexamer |
| Nucleation with hexamer | 3*Dimer + Hexamer -> 2*Hexamer |
| Nucleation with chaperone and hexamer | 3*Dimer + ChapHexamer -> Hexamer + ChapHexamer |
| Chaperone-hexamer formation | 3*ChapDimer -> ChapHexamer |
| Chaperone-hexamer binding | Hexamer + 6*Chap -> ChapHexamer |
| Chaperone (with hexamer) release | ChapHexamer -> 6*Chap + Hexamer |
| Hexamer refolding | ChapHexamer + Hsp104 -> 6*Chap + 6*ProtF + Hsp104 |
| Cln3 binding | Chap + Cln3 -> ChapCln3 |
| Chaperone-Cln3 unbinding | ChapCln3 -> Chap + Cln3 |
| Protein synthesis | Φ -> Prot |
| Chaperone synthesis | Φ -> Chap |
| Cln3 synthesis | Φ -> Cln3 |
| Unfolded protein degradation | Prot -> Φ |
| Folded protein degradation | ProtF -> Φ |
| Misfolded protein degradation | ProtM -> Φ |
| Dimer degradation | Dimer -> Φ |
| Hexamer degradation | Hexamer -> Φ |
| Chaperone degradation | Chap -> Φ |
| Cln3 degradation | Cln3 -> Φ |
| Cln3 (nuclear) degradation | Cln3Nuc -> Φ |
| Protein (with chaperone) degradation | ChapProt -> Chap |
| Chaperone (with protein) degradation | ChapProt -> Prot |
| Misfolded protein (with chaperone) degradation | ChapM -> Chap |
| Chaperone (with misfolded protein) degradation | ChapM -> ProtM |
| Dimer (with chaperone) degradation | ChapDimer -> 2*Chap |
| Chaperone (with dimer) degradation | ChapDimer -> Dimer |
| Hexamer (with chaperone) degradation | ChapHexamer -> 6*Chap |
| Chaperone (with hexamer) degradation | ChapHexamer -> Hexamer |
| Cln3 (with chaperone) degradation | ChapCln3 -> Chap |
| Chaperone (with Cln3) degradation | ChapCln3 -> Cln3 |
| Whi5 inactivation | Whi5 + Cln3Nuc -> Whi5i + Cln3Nuc |
| Whi5 activation | Whi5i -> Whi5 |

^1^ Species are: Prot - Unfolded protein; Chap - Chaperone; ChapProt - Chaperone-Unfolded protein complex; ProtF - Folded protein; ProtM - Misfolded protein; Dimer - Dimer of misfolded proteins; Hexamer - Nucleated hexamer of misfolded proteins; ChapHexamer - Chaperone-Hexamer complex; ChapM - Chaperone-Misfolded protein complex; ChapDimer - Chaperone-Dimer complex; Hsp104 - Hsp104 protein ; Cln3 – Unfolded ER bound Cln3; ChapCln3 - Chaperone-Cln3 complex; Cln3Nuc - Nuclear Cln3; Whi5 - Active Whi5; Whi5i - Inactive Whi5; ø - No reactants/products
