# Supplementary material for: Proteostasis collapse, a hallmark of aging, hinders the chaperone-Start network and arrests cells in G1
Source: eLife. 2019 Sep 13;8:e48240. doi: 10.7554/eLife.48240 (PMC6744273; doi:10.7554/eLife.48240)
Supplement: Supplementary file 2. [file elife-48240-supp2.docx]

**Supplementary file 2.** Parameter set of the integrative mathematical model.

| **Reaction name** | **Parameter value and units** |
| --- | --- |
| Unfolded protein-chaperone binding | 10^9^ fl*fmol^-1^*s^-1^ |
| Protein folding | 3 s^-1^ |
| Protein misfolding | 0.078 s^-1^ |
| Misfolded protein refolding | 1 s^-1^ |
| Chaperone-misfolded protein binding | 10^10^ fl*fmol^-1^*s^-1^ |
| Dimerization (fast) | 10^7^ fl^2^*fmol^-2^*s^-1^ |
| Dimerization (slow) | 3*10^5^ fl*fmol^-1^*s^-1^ |
| Dimerization with hexamer | 10^15^ fl^2^*fmol^-2^*s^-1^ |
| Dimerization with chaperone and hexamer | 10^7^ fl^2^*fmol^-2^*s^-1^ |
| Chaperone-dimer binding | 6*10^18^ fl^2^*fmol^-2^*s^-1^ |
| Dimer refolding | 10^-3^ fl^3^*fmol^-3^*s^-1^ |
| Nucleation | 5*10^8^ fl^2^*fmol^-2^*s^-1^ |
| Nucleation with hexamer | 10^23^ fl^3^*fmol^-3^*s^-1^ |
| Nucleation with chaperone and hexamer | 10^18^ fl^3^*fmol^-3^*s^-1^ |
| Chaperone-hexamer formation | 10^10^ fl^2^*fmol^-2^*s^-1^ |
| Chaperone-hexamer binding | 10^55^ fl^6^*fmol^-6^*s^-1^ |
| Chaperone (with hexamer) release | 10^-5^ s^-1^ |
| Hexamer refolding | 10^4^ fl*fmol^-1^*s^-1^ |
| Cln3 binding | 10^9^ fl*fmol^-1^*s^-1^ |
| Chaperone-Cln3 unbinding | 3 s^-1^ |
| Protein synthesis | 10^-5^ fl*fmol^-1^*s^-1^ |
| Chaperone synthesis | 9*10^-7^ fl*fmol^-1^*s^-1^ |
| Cln3 synthesis | 10^-7^ fl*fmol^-1^*s^-1^ |
| Unfolded protein degradation | 1 s^-1^ |
| Folded protein degradation | 1 s^-1^ |
| Misfolded protein degradation | 10^-1^ s^-1^ |
| Dimer degradation | 10^-2^ s^-1^ |
| Hexamer degradation | 10^-3^ s^-1^ |
| Chaperone degradation | 1 s^-1^ |
| Cln3 degradation | 15 s^-1^ |
| Cln3 (nuclear) degradation | 1 s^-1^ |
| Protein (with chaperone) degradation | 1 s^-1^ |
| Chaperone (with protein) degradation | 1 s^-1^ |
| Misfolded protein (with chaperone) degradation | 5 s^-1^ |
| Chaperone (with misfolded protein) degradation | 1 s^-1^ |
| Dimer (with chaperone) degradation | 10^-1^ s^-1^ |
| Chaperone (with dimer) degradation | 1 s^-1^ |
| Hexamer (with chaperone) degradation | 10^-3^ s^-1^ |
| Chaperone (with hexamer) degradation | 1 s^-1^ |
| Cln3 (with chaperone) degradation | 15 s^-1^ |
| Chaperone (with Cln3) degradation | 1 s^-1^ |
| Whi5 inactivation | 2.1*10^8^ fl*fmol^-1^*s^-1^ |
| Whi5 activation | s^-1^ |
