# Supplementary material for: Proteostasis collapse, a hallmark of aging, hinders the chaperone-Start network and arrests cells in G1
Source: eLife. 2019 Sep 13;8:e48240. doi: 10.7554/eLife.48240 (PMC6744273; doi:10.7554/eLife.48240)
Supplement: Supplementary file 3. [file elife-48240-supp3.docx]

**Supplementary file 3.** Parameter modifications to simulate different genotypes or relevant physiological conditions.

| **Simulation** | **Parameter alterations** | **Initial size** |
| --- | --- | --- |
| Medium cell volume | None | 24.8fl (same as wt) |
| Large cell volume | None | 39.0fl |
| Small cell volume | None | 17.1fl |
| Fast growth | Increase growth and all synthesis rates by 10% | 24.8fl (same as wt) |
| Slow growth | Decrease growth and all synthesis rates by 10% | 24.8fl (same as wt) |
| ydj1 | Decrease synthesis rate of chaperone by 1% | 36.3fl |
| oYDJ1 | Increase synthesis rate of chaperone by 1% | 23.6fl |
| cln3 | Decrease synthesis rate of Cln3 by 25% | 39fl |
| oCLN3 | Increase synthesis rate of Cln3 by 10% | 17.1fl |
| whi5 | Decrease Whi5 concentration by 10% | 18.5fl |
| oPFD | Increase protein misfolding rate by 10% | 24.8fl (same as wt) |
| oPFD oYDJ1 | Increase protein misfolding rate by 10%  Increase synthesis rate of chaperone by 1% | 23.6fl |
| oPFD oCLN3 | Increase protein misfolding rate by 10% Increase synthesis rate of Cln3 by 10% | 17.1fl |
| ydj1 oCLN3 | Decrease synthesis rate of chaperone by 1% Increase synthesis rate of Cln3 by 10% | 17.1fl |
